# Decentralisation in Times of Crisis: Asset Or Liability? The Case of Germany and Italy During Covid‐19

**DOI:** 10.1111/spsr.12482

**Published:** 2021-10-08

**Authors:** Katharina Kuhn, Irene Morlino

**Affiliations:** ^1^ London School of Economics and Political Science

## Abstract

How did the legal and political‐administrative relationship between central and local governments of two decentralised states shape their response to COVID‐19? Literature and theories on decentralisation argue that federal and decentralised states are less able to respond to crises in a coordinated manner due to their perceived greater susceptibility to political conflict. Situated within this theoretical debate and based on the analysis of legal acts, political decisions, and relevant national news media articles between March and August 2020 in Germany and Italy, this research note shows that, counterintuitively, more decentralisation does not necessarily translate into more legal and political stress during pandemic management. In responding to the COVID‐19 pandemic, Germany, a highly decentralised state, experienced less legal and political tensions than the less decentralised Italy. The key to understanding this variation lies in different institutional arrangements, complemented by the specific political cultures of both states.

## Zusammenfassung

Wie beeinflussten die rechtlichen und politisch‐administrativen Beziehungen zwischen lokalen und zentralen Regierung zweier dezentraler Staaten deren Umgang mit COVID‐19? In der Literatur über Dezentralisierung gelten föderale und dezentralisierte Staaten aufgrund ihrer potentiell höheren Anfälligkeit für politische Konflikte als weniger in der Lage, koordiniert auf Krisen zu reagieren. Eingebettet in diese theoretische Debatte und basierend auf der Analyse von rechtlichen Verordnungen, politischen Entscheidungen und relevanten nationalen Nachrichtenartikeln zwischen März und August 2020 in Deutschland und Italien zeigt die vorliegende Research Note, dass mehr Dezentralisierung entgegen diesen Erwartungen nicht zwangsläufig zu mehr rechtlichem und politischem Stress während des Managements einer Pandemie führen muss. Als Reaktion auf die COVID‐19‐Pandemie erlebte Deutschland, ein stark dezentralisierter Staat, weniger rechtliche und politische Spannungen als das weniger dezentralisierte Italien. Der Schlüssel zum Verständnis dieser Unterschiede sind die unterschiedlichen institutionellen Arrangements der jeweiligen Länder, ergänzt durch ihre spezifischen politischen Kulturen.

## Résumé

Comment la relation juridique et politico‐administrative entre les gouvernements centraux et locaux de deux États décentralisés a‐t‐elle modelé leur réponse au COVID‐19 ? La littérature et les théories sur la décentralisation soutiennent que les États fédéraux et décentralisés sont moins capables de répondre aux crises de manière coordonnée en raison de leur plus grande prédisposition aux conflits politiques. Cette note de recherche contribue à ce débat théorique par une analyse des actes juridiques, des décisions politiques et des articles de la presse nationale entre mars et août 2020 en Allemagne et en Italie, qui montre que, contre toute attente, plus de décentralisation n’a pas nécessairement conduit à plus des tensions juridiques et politiques lors de la gestion de la pandémie. En réponse au COVID‐19, l'Allemagne, un État très décentralisé, a connu moins de tensions juridiques et politiques que l'Italie, moins décentralisée. La clé pour comprendre cette variation réside dans les différents arrangements institutionnels, complémentés par les cultures politiques spécifiques des deux États.

## INTRODUCTION

When the COVID‐19 crisis erupted, many states had to take unprecedented health measures to contain the spread of the virus. The pandemic defied domestic political and legal conventions, especially in decentralised countries characterised by the distribution of political and legal power and competences between a central government and constituent units. Germany and Italy represent two particularly distinctive examples along the spectrum of decentralisation. Germany is a federal state characterised by constituent units (*Länder*), while Italy is a decentralised state composed of regions (*regioni*). Situated within theoretical debates on decentralisation and based on a content and discourse analysis of national news media articles between March and August 2020, this research note asks: *how did the specific legal and political‐administrative relationship* between central and local governments *of Germany and Italy shape their management of the COVID‐19 crisis?* Federal and decentralised states are thought to be prone to ineffective responses due to the risk of high levels of political conflict and hence less able to coordinate their crisis response. Counterintuitively, our research note shows that Germany, a highly decentralised country, experienced significantly less legal and political stress during pandemic management than the less decentralised Italy. We argue that this is due to different institutional arrangements complemented by some features of intra‐ and inter‐governmental political culture. First, we consider the relevant theoretical and empirical literature on decentralization and federalism before turning to a brief discussion of our methodology. Following on from this discussion, we then argue, with supporting empirical evidence, that the legal and political‐administrative relationship between different levels of government in Germany and Italy impacted crisis management in both states. Finally, we consider the comparability of our empirical findings and their applicability for future work.

## DECENTRALISATION AND EMERGENCY

Studies on decentralisation are numerous and have analysed different issue areas, such as health, education, economic and fiscal stability, development (Abimbola et al., 2019; Costa‐i‐Font & Greer, 2013; Madon et al., 2010; Saltman & Bankauskaite, 2006; Stegarescu, 2005; Rondinelli, 1983). Other studies have focussed on the effects of decentralisation on political and fiscal governance and accountability (Fauget, 2014; Wibbels, 2005; Oxhorn et al., 2004; Bardhan, 2002; Mello & Barenstein, 2001).

Decentralisation refers to the reallocation of power from higher/central to lower/constituent levels and can be of different types: political, administrative, or fiscal (Benz, 2011; Treisman, 2007). For the purposes of this research note, we consider political decentralisation to be the shift of policy‐making responsibility from the central government to local/constituent units within a country (Pollitt, 2005). This definition involves the delegation of both legislative and executive powers from the central government to the local/constituent units, which are accountable to the electorate (Hermansson, 2019). Thus, political decentralisation is reflected in a particular legal and political‐administrative relationship between different levels of government. We define the *legal relationship* between government levels as the interaction among central and local authorities based on the allocation of legal authority and competencies to the centre and the constituent units as defined by the constitution and legal provisions (Costa‐i‐Font & Greer, 2013). The laws governing decentralisation shape the *political‐administrative relationship* between different levels of government, since power is constitutionally allocated in such a way that the different levels of government can legislate independently (Bolleyer & Thorlakson, 2013). This relationship becomes politically relevant, since it constitutes the basis for the discursive attribution of, and claims to, management responsibilities and competencies by political stakeholders at different levels of government. Competing claims or shifts of responsibility by political stakeholders may impede swift and effective policy responses to a crisis due to a variety of mechanisms.

Decentralisation allows for the settling of political tensions, guaranteeing closer contact between government representatives and their citizens, which better reflects the plurality and regional differentiation of social interests (Benz, 2011; Paquin, 2011; Conyers, 2000). However, decentralisation can also increase the risk of conflict among a plurality of actors all seeking to hold/share power, thereby potentially causing disputes between different levels of government over the management of resources (Shou & Haug, 2005). Indeed, because of their institutional complexity, federal and decentralised states are generally considered to be less able to respond to crises in a rapid and coordinated manner (Hegele & Schnabel, 2021). As a result of decentralization, individual actors might have the incentive to engage in a ‘blame game’, wherein all actors attempt to avoid responsibility for potentially risky or unpopular decisions (Birkland & Waterman, 2008; Schneider, 2008). This is particularly relevant when an emergency or crisis occurs, exacerbating existing and complex dynamics deriving from decentralisation (Colan, 2006; Carter & May, 2020; Migone, 2020a, 2020b). Finally, decentralised states can be prone to ineffective political responses to a crisis if they lack clear and effective political and legal guidelines for the coordination of different levels of government (Gerber & Robinson, 2009; Landy, 2008; Menzel, 2006). Hence, the specific existing legal and political‐administrative relationship between different levels of government may impact the ability of a government to effectively handle exogenous shocks such as the COVID‐19 pandemic.

## METHODOLOGY

To address our research question, we focus on Germany and Italy from March to August 2020. Among all members of the European Union, these two countries represent most different cases and diverge on several important characteristics. Germany is among the most populous democracies in Western Europe. Over the last 15 years, it has been the most resilient European country in the wake of the 2008 economic crisis, with a constant national surplus and stable government.^1^ Contrastingly, Italy has struggled severely, with seven years of stagnation, a very high national public debt and government instability.^2^ Germany and Italy also occupy different positions on a continuum of decentralisation: Germany is a federal state and Italy is a decentralised one (Paquin, 2011). As a result, this research note compares the COVID‐19 pandemic responses of two countries that were affected by the same crisis but differ both in their degree of decentralisation and in their socio‐economic characteristics. In doing so, we intend to show how the legal and political‐administrative relationship of these two countries shaped their ability to manage the COVID‐19 crisis. We assume that the specific configuration of the legal and political‐administrative relationship acts as an intervening variable that impacts the management capability (dependent variable) of a given state during a crisis (independent variable).

Our analysis is limited to the ‘first wave’ of the pandemic (March‐August 2020) and is based on the analysis of legal acts, political decisions, and the relevant national newspaper articles.^3^ First, we conducted a content analysis to understand whether and how the pandemic impacted the legal relationship between different levels of government, i.e. whether and how the COVID‐19 pandemic impacted the distribution of legal competencies between different levels of government. To understand whether and to what degree this was the case, we relied on relevant legal texts that described changes in the legal relationship between the central government and its constituent units. Second, we conducted a discourse analysis of government discourses and political decisions reported in news media to understand how political stakeholders framed their relationship with each other and therefore how COVID‐19 impacted the political‐administrative relationship between different levels of government. In particular, our analysis is based on statements made by politicians belonging to different levels of government about the political responsibility for crisis management and about the relationship between the constituent units and the central government. Such statements, for instance, advocate for coordination, shift blame, or claim sole responsibility for adopting or easing measures.

## GERMANY

The German Basic Law does not provide for the centralisation of authority in the case of an emergency. In contrast, emergency management is a competency of the *Länder* (Pohlmann, 2013: 252). Pandemic management as part of health policy is part of concurrent legislation under Article 74 para 1 no 19 of the German Basic Law. This means that the *Länder* have legislative power unless the federal government uses its concurrent powers. With the adoption of the first Infection Protection Law (IfSG) by the *Bundestag* in 2000, the federal government used these powers (Wissenschaftliche Dienste des Deutschen Bundestags, 2020a: 4). The implementation of the IfSG through the adoption of measures such as lockdown and contact restrictions are the competency of the *Länder* (Kießling, 2020).

The COVID‐19 pandemic did not impact the legal relationship between the federal government and the *Länder* insofar as the clear division of competencies for both healthcare and emergency management was maintained^4^ in line with the overall tradition of ‘executive federalism’. Executive federalism describes “a functional separation under which the federal government is assigned the bulk of legislative power while the states exercise most administrative powers” (Heidenheimer, 1966: 172 as cited in Rudzio, 2019: 307). In line with this separation of legal competencies, the *Länder* adopted their own lockdown and contact restrictions to implement the IfSG. The federal level provided the legislative framework for pandemic management when, on 25 March 2020, the *Bundestag* declared the existence of a nationwide pandemic situation based on § 5 para 1 sentence 1 IfSG, and subsequently applied the IfSG (Wissenschaftliche Dienste des Deutschen Bundestags 2020b: 4). This division of legal competencies between *Bund* and *Länder* was further maintained in both amendments to the IfSG that were adopted on 27 March 2020^5^ and 19 May 2020,^6^ respectively. Following the amendments, § 5 para 2 grants the federal Ministry of Health further executive powers but does so “without prejudice to the powers of the *Länder*”.

The political‐administrative relationship of *Bund* and *Länder* during COVID‐19 mirrored the German tradition of unitary federalism that is marked by a cooperative political culture and high levels of coordination (Braun 2003; Hüttmann 2010). During non‐crisis times, coordination between the heads of *Bund* and *Länder* as well as their ministries, including the respective ministries of health, takes place through regular informal meetings (Gebauer, 2006: 131). Although the agreements reached in these consultations are non‐binding, *Bund* and *Länder* usually abide by them. Therefore, even issues that fall under the exclusive competence of the *Länder* are marked by high levels of standardisation and harmonisation (Rudzio, 2019: 313f.).

Especially during the first months of the pandemic – from the first measures in March until the introduction of a regional threshold in May 2020 – both the federal government and the *Länder* relied heavily on cross‐government coordination. On 12 March, Chancellor Merkel and the presidents of the *Länder* held their first joint consultation to discuss the appropriate steps for pandemic management and agreed that “the federal government and the Länder will closely cooperate in handling the pandemic” (Presse‐ und Informationsamt der Bundesregierung 2020). While the *Länder* used their executive powers to adopt measures independently from each other, they did so within this non‐binding framework of joint coordination. The deviation of several *Länder* from the jointly agreed guidelines in easing lockdown restrictions were strongly condemned by federal and other *Länder* politicians in favour of slower steps (Schröder, 2020; dpa, 2020). In May, the attribution of political‐administrative responsibilities changed, when the *Länder,* together with Chancellor Merkel, agreed on a threshold of 50 new infections per 100,000 inhabitants as a key tool for pandemic management. Although the legal relationship between *Bund* and *Länder* remained unchanged by this decision, mainstream media framed the introduction of this mechanism as a symbolic “delegation of (political) responsibility” from the federal to the state level (Mestermann & Schröder, 2020). Although Merkel continued to call for coordination between the *Länder*, the latter stressed their individual responsibility and adopted greatly diverging strategies in the following weeks: while Thüringen and Brandenburg lifted all contact restrictions two weeks before the agreed end‐date, Bavaria introduced additional tests for tourists returning from their holidays (Groll, 2020; Günther, 2020; Endres, 2020). The prospect of elections in several *Länder*, the federal election in 2021, and upcoming internal elections in the CDU/CSU intensified these differences, as the presidents of several *Länder* used debates about pandemic management strategies to build their distinct names and gain consensus among their constituencies. This may explain the political competition on easing lockdown restrictions between Markus Söder (Bavaria) and Achim Laschet (Nordrhein‐Westfalen), which media commentators linked to both politicians’ aspirations to be nominated as candidate for the chancellorship by the CDU/CSU (Schnell, 2020; Rohr, 2020).

By emphasising unity and strategic coordination, the governments of the *Länder* and the federal government attributed the political responsibility for pandemic management to both levels of government as well as to the collective of all *Länder*. Thereby, they emphasised their joint responsibility, and the governments of the *Länder* could divert the political risks of pandemic management to other stakeholders. This logic became visible from May 2020 onwards, when the *Länder* began adopting diverging strategies: *Länder* that experienced higher overall numbers and a quicker rise of new cases in August feared that an earlier end of lockdown restrictions by other *Länder* would relativise the severity of the situation and endanger their own positive developments. Hence, they called for a renewed stronger role of federal coordination (Glas, 2020; Maxwill, 2020; Ludwig, 2020). *Länder* with lower numbers of cases, on the contrary, employed the key rationale of federalism – the ability to account for regional differences – in order to justify their quicker pace in easing restrictions (Höhne, 2020). This suggests that the *Länder* used coordination between *Bund* and *Länder* as a ‘backstop’ to increased uncertainty and to collateralise the political risks of decision‐making in highly volatile situations: the *Länder* endorsed coordination especially when policy making was risky and decisions were potentially unpopular, e.g. at the beginning and at the end of the first wave of the pandemic. In both situations, the merits of different pandemic management strategies were still unclear. By building on traditions of unitary federalism and by aligning their measures with joint guidelines, the *Länder* enjoyed the ability to divert the responsibility to other stakeholders in case of failure. At the same time, similar actions in other *Länder* validated individual strategies. The political‐administrative relationship between *Bund* and *Länder* as well as among the *Länder* can hence be described as a ‘pre‐emptive blame game’ in which political stakeholders stressed joint responsibility in order to maintain the possibility of horizontally shifting responsibility (and, if necessary, blame) to the collective of the *Länder*.

## ITALY

According to art. 117 of the Italian Constitution, healthcare is a shared competence between the state and the regions. However, in case of “severe danger to public safety and security” Art. 120 allows the government to supersede regions in addressing public concerns. In addition to Art. 120, other measures such as law n° 833, adopted in 1978, grant the President of the Council and the Minister of Health the right to intervene in the case of a pandemic. Furthermore, regional authorities can also take provisions in their territory when they deem it necessary.^7^

COVID‐19 had an impact on both the legal and political‐administrative relationships between the state and the regions. The content and discourse analyses conducted of the legal acts, political decisions, and the relevant national newspaper during the first wave of the pandemic (March 2020 to August 2020) demonstrate that the pandemic further exacerbated existing legal and political conflicts between the state and the regions.

Concerning the legal relationship, the principle of loyal cooperation established by the Constitutional Court^8^ is a tenet that should govern relations between state and regions in those fields where their competencies concur and intersect (Bin & Pitruzzella, 2011: 220). However, legal provisions for healthcare were insufficiently clear to guarantee an effective long‐term response. When the pandemic hit Italy, both state and regions struggled to share healthcare competencies. This resulted in an asymmetry in the measures adopted at the national level and several legal conflicts over respective healthcare competencies (Clementi, 2020). In particular, the state appeared weaker than might have been expected, allowing the presidents of the regions to adopt legal measures that overlapped and contradicted policies put forward by the state. During the first phase (March to April 2020), the Agency for Civil Protection, which is responsible for risk prevention and intervention following an emergency, together with the Ministry of Interior released a legal order to harmonise and overcome the many different provisions taken by regional administrations during the first weeks of the pandemic. Indeed, the regions had already taken more restrictive measures than those of the government (Mirabelli, 2020).

The legal contrasts between these two different levels of government characterised Italy’s response between March and April 2020. They subsided only when the central government released a decree renouncing its coordination and harmonisation role, thus, allowing each region to introduce expansionary and restrictive measures autonomously. The central government would however maintain a supervisory function: if infections were to rise, it would retain the ability to intervene at the regional level (Il Sole 24 Ore, 2020a).

In addition, the content and discourse analyses undertaken highlight how COVID‐19 specifically impacted the political‐administrative relationship between state and regions. These sources also demonstrate how the crisis led to a political and vertical ‘blame game’ between the two different government levels: neither the president of the Council, Giuseppe Conte, nor the presidents of the *regioni* were willing to take unpopular political decisions. One relevant example of the ‘blame game’ evident in Italy’s response to the Covid‐19 pandemic is the case of Lombardy. At the beginning of the pandemic, the president of Lombardy, Attilio Fontana, waited for the Council President’s lockdown decree of 7‐8 March rather than taking swift measures. Fontana had an ambiguous approach: on the one hand, he was aware of the urgency of closing the Bergamo area and had previously urged for more autonomy from the state to shut down earlier (Gabbanelli & Ravizza, 2020); on the other hand, as a judicial investigation has shown (Franco, 2020; Il Sole 24 Ore, 2020b), Lombardy’s local authorities, including Fontana, were put under pressure by industrial lobbies to avoid closures. Lombardy is in fact one of the most industrialised areas of Europe and industrial lobbies have a significant influence on local politics. Therefore, Fontana did not take action, later blaming the central government action for not being prompt and effective. As the Council president said, Fontana could have pre‐empted the government’s action by taking measures to contain viral spread, just as the *regioni* of Veneto, Lazio and Campania had done by circumscribing ‘red zones’. On the contrary, Fontana continued to argue that it was the government’s responsibility to take the restrictive measures (Ainis, 2020; Greco, 2020). Thus, the COVID‐19 crisis highlights an important issue: in the face of a relatively unpopular decision, all actors (including the central government and the *regioni*’s authorities) refuse to take political responsibility for potentially costly health measures. Indeed, Fontana refused to take restrictive measures to avoid losing political consensus and support of his electorate, including local industries (Trocino, 2020). As such, there is some evidence to support the notion that blame shifting dominated politicians’ and parties’ considerations in the face of potentially unfavourable returns at the ballot box.

The impact of COVID‐19 on the political‐administrative relationship between governance levels becomes evident in the changing role of the presidents of the regions. Since the beginning of the pandemic, they became much more relevant figures at the centre of political decisions and of media attention, shadowing their respective parties. This marked a further trend towards “personalisation” and, consequently, of “presidentialization” (Calise, 2020; Diamanti, 2020) of the Council president, Conte, as well as the *regioni* presidents, and particularly those of Veneto, Lombardy, Emilia‐Romagna, and Campania. The media emphasised that they played a central role in managing the pandemic. Furthermore, their popularity increased among regional electorate. In April of 2020, 42% of Italians stated that they thought that the regions had performed better in managing the Covid‐19 pandemic than the central government, while 34% said the opposite (Pagnoncelli, 2020a, 2020b). This resulted also in an increased popularity of the parties they represented, which opposed the Five Star Movement and, hence, central government led by Conte. The link between the regions’ presidents and their parties came across in many instances. For instance, Zaia and Fontana, who belonged to the League, were openly supported by Salvini who often made statements strengthening or anticipating the positions taken by the two regions’ presidents (La Stampa, 2020). Finally, the increased popularity among the electorate strengthened the claim of Veneto, Emilia‐Romagna and Lombardy for “differentiated autonomy”, i.e. greater autonomy from the state, a request that the regions had already put forth in 2018.

## CONCLUSION

Our research note has sought to understand *how the specific legal and political‐administrative relationship* between central and local governments *of Germany and Italy shaped the management of the COVID‐19 crisis in both countries*. Based on the analysis of legal acts, political decisions, and relevant national news media articles, we show that, in both countries, the pandemic further accentuated already existing dynamics in the legal and political‐administrative relationship between central government and constituent units. In Germany, COVID‐19 left the legal and political‐administrative relationship between *Bund* and *Länder* unscathed, thanks to an established legal division of competencies for both healthcare and emergency management in line with the tradition of ‘executive federalism’. In addition, despite the pandemic, the political‐administrative relationship in Germany followed a pattern of unitary federalism, which is marked by high levels of cooperative political culture and coordination. In contrast, COVID‐19 further exacerbated Italy’s existing legal and political conflicts between central state and *regioni*. The legal provisions for governing healthcare were not clear enough to guarantee an effective long‐term response, and state and regions struggled in sharing healthcare competencies. This led not only to asymmetrical measures adopted across the country, but also impacted the political‐administrative relationship between government levels. The crisis produced a vertical political ‘blame game’, by which neither the president of the Council nor the presidents of the regions were willing to take unpopular decisions and bear responsibility for a possible failure of the measures. The crisis also strengthened the role of the regional presidents who, for the first time, became key figures, attracting media attention and gaining increasing popularity and notability among the electorate. Conversely, in Germany, the shifting of responsibility for the introduction of health measures took place predominantly horizontally: by coordinating measures with joint guidelines during times of high uncertainty and political risk, the presidents of the *Länder* maintained the possibility of shifting blame to other stakeholders (such as the collective of all *Länder*) in case of failure, thereby embarking on a ‘pre‐emptive blame game’.

From a theoretical perspective, federal and decentralised states are expected to be less able to respond to a crisis in a coordinated way. However, this comparison between Germany and Italy shows that Germany, a highly decentralised country, experienced much less legal and political stress during its pandemic management than the less decentralised Italy. Our analysis indicates that is largely due to a long history of coordination and cooperation among constituent units and a definite legal division of competencies among the German *Länder* and the federal government. In contrast, in Italy, the pandemic deepened and further exacerbated the contrasts between state and regions because of an already existing indeterminacy of competencies and overlaps of legal measures, which paved the way for a political ‘blame game’, creating confusion and ineffectiveness. This suggests that the degree of decentralisation that shapes a country’s ability to respond to a crisis, such as a pandemic, is less crucial than the specific configuration of the legal and political‐administrative relationship between the central government and the constituent units.

## Data Availability

The data that support the findings of this study are available from the corresponding author upon reasonable request.

## Footnotes

^1^
See World Bank for unemployment https://data.worldbank.org/indicator/SL.UEM.TOTL.ZS?end=2020&locations=DE‐IT‐GB‐ES‐FR&start=2008&view=chart; https://databank.worldbank.org/reports.aspx?source=2&series=NY.GDP.MKTP.KD.ZG&country=DEU,ITA,FRA,ESP,GBR; economic growth https://databank.worldbank.org/reports.aspx?source=2&series=NY.GDP.MKTP.KD.ZG&country=DEU,ITA,FRA,ESP,GBR; poverty, https://data.worldbank.org/indicator/SI.POV.NAHC?end=2018&locations=DE‐IT‐FR‐GB‐ES&start=2008&view=chart; see Eurostat for national debt: https://ec.europa.eu/eurostat/statistics‐explained/index.php?title=File:General_government_debt,_2018_and_2019_(%C2%B9)_(General_government_consolidated_gross_debt,_%25_of_GDP)_2020‐10‐22.png; social protection, https://ec.europa.eu/eurostat/web/social‐protection/data/database; health, https://ec.europa.eu/eurostat/web/health/data

^2^
See European Central Bank data: https://sdw.ecb.europa.eu/reports.do?node=1000004191

^3^
Germany: Der Spiegel, Frankfurter Allgemeine Zeitung, Spiegel Online, Zeit, Zeit Online, Süddeutsche Zeitung, Süddeutsche Zeitung Online; Italy: Corriere della Sera, Il Giornale, Il Mattino, Il Messaggero, Il Sole 24 Ore, La Repubblica, La Stampa.

^4^
Note that this refers to our period of investigation (March – August 2020) only.

^5^

*Erstes Gesetz zum Schutz der Bevölkerung bei einer epidemischen Lage von nationaler Tragweite*, see Bundesgesetzblatt 14/2020, pp.587ff. https://www.bgbl.de/xaver/bgbl/start.xav?startbk=Bundesanzeiger_BGBl&jumpTo=bgbl100s1045.pdf#__bgbl__%2F%2F*%5B%40attr_id%3D%27I_2020_14_inhaltsverz%27%5D__1614013775170.

^6^

*Zweites Gesetz zum Schutz der Bevölkerung bei einer epidemischen Lage von nationaler Tragweite*, see Bundesgesetzblatt 23/2020, pp.1018ff. https://www.bgbl.de/xaver/bgbl/start.xav?startbk=Bundesanzeiger_BGBl&jumpTo=bgbl100s1045.pdf#__bgbl__%2F%2F*%5B%40attr_id%3D%27I_2020_23_inhaltsverz%27%5D__1614013984568

^7^
Law 23 December 1978 n. 833 (comma 6b).

^8^
Judgement 242/1997.
